# Interconnected mental health symptoms: network analysis of depression, anxiety, stress, and burnout among psychiatric nurses in the context of the COVID-19 pandemic

**DOI:** 10.3389/fpsyt.2024.1485726

**Published:** 2024-10-28

**Authors:** Rui Tao, Song Wang, Qingfang Lu, Yunxiao Liu, Lei Xia, Daming Mo, Feng Geng, Tingfang Liu, Yuanli Liu, Feng Jiang, Huan-Zhong Liu, Yi-lang Tang

**Affiliations:** ^1^ Department of Psychiatry, Chaohu Hospital of Anhui Medical University, Hefei, China; ^2^ Department of Psychiatry, School of Mental Health and Psychological Sciences, Anhui Medical University, Hefei, China; ^3^ Department of Psychiatry, Anhui Psychiatric Center, Hefei, China; ^4^ Department of Psychiatry and Psychology, Taihe Hospital of Traditional Chinese Medicine, Fuyang, China; ^5^ Department of Substance-Related Disorders, Hefei Fourth People’s Hospital, Hefei, China; ^6^ Department of Psychiatry, Second Affiliated Hospital of Anhui Medical University, Hefei, China; ^7^ Research Department, School of Health Policy and Management, Chinese Academy of Medical Sciences & Peking Union Medical College, Beijing, China; ^8^ Research Department, School of International and Public Affairs, Shanghai Jiao Tong University, Shanghai, China; ^9^ Research Department, Institute of Healthy Yangtze River Delta, Shanghai Jiao Tong University, Shanghai, China; ^10^ Institute of Health Policy, Shanghai Jiao Tong University, Shanghai, China; ^11^ Institute of Grand Health, Wenzhou Medical University, Wenzhou, China; ^12^ Department of Psychiatry, Huizhou Second People’s Hospital, Huizhou, China; ^13^ Addiction Psychiatry Fellowship Program, Department of Psychiatry and Behavioral Sciences, Emory University, Atlanta, GA, United States; ^14^ Mental Health Service Line, Joseph Maxwell Cleland Atlanta VA Medical Center, Decatur, GA, United States

**Keywords:** network analysis, burnout, stress, depression, anxiety, psychiatric nurses

## Abstract

**Background:**

Mental health symptoms such as anxiety, depression, stress, and burnout are common among healthcare workers. However, the interconnections among them remain under-explored. This study aimed to address the interrelationships among these symptoms in psychiatric nurses.

**Methods:**

We conducted a nationwide survey in the early stage of the COVID-19 pandemic (January to March 2021) to investigate the interconnectedness of depression, anxiety, stress, and burnout among psychiatric nurses. Using network analysis, we identified central symptoms, important bridge symptoms, and the correlations among these central symptoms.

**Results:**

Of the 9,224 psychiatric nurses (79.2% female) included in the statistical analyses, 27.6% reported clinically significant depression, 31.2% anxiety, 14.5% stress, and 23.8% burnout. Network analysis revealed that stress had the highest expected influence (EI) value (0.920) and the highest strength among all nodes. The node for depression scored the highest in both closeness and betweenness. Emotional exhaustion (EE) had the highest bridge expected influence (BEI) of 0.340, with the strongest intergroup association between EE and depression. No significant differences were found in gender or frontline work experience (all p > 0.05).

**Conclusions:**

Burnout, depression, anxiety, and stress are relatively common among psychiatric nurses in the context of the COVID-19 pandemic. While anxiety was the most prevalent, stress emerged as the core symptom, and depression as an important bridging node. Interventions targeting the core symptoms and bridging nodes may improve the mental health of psychiatric nurses.

## Introduction

The COVID-19 pandemic has significantly impacted global public health and mental health systems (1, 2), causing a surge in global adult mortality rates in 2020 and 2021 (3, 4). A global survey estimated that there were 53.2 million more cases of major depressive disorder globally (a 27.6% increase) and 76.2 million more cases of anxiety disorders globally (a 25.6% increase) due to the pandemic (5). Healthcare workers, particularly nurses, have been disproportionately affected, experiencing higher rates of depression, anxiety, stress, insomnia, and burnout (6–12). In France, a nationwide online survey conducted during the pandemic found that 55.2% of 10,087 healthcare workers reported burnout, while 64.8% reported poor sleep (6). These surveys indicate that the pandemic has had a significant and serious impact on the physical and mental well-being of various populations, particularly healthcare workers (13–17). As crucial components of healthcare practitioners, nurses often work on the front line of patient care and are also vulnerable to developing mental health problems, including helplessness, depression, anxiety, stress, and job burnout (18, 19), which are particularly common in China (20–22). A survey in China involving 138,279 nurses from 243 hospitals showed that a substantial proportion of nurses reported experiencing symptoms of burnout (34%), anxiety (41.8%), and depression (55.5%) (20). An interrupted time-series analysis of preliminary data from 38 countries has also shown similar findings (23).

While numerous studies have documented the prevalence of mental health problems among nurses during the pandemic, there is a critical gap in understanding the underlying mechanisms and interrelationships between these issues. Traditional approaches have been limited in their ability to elucidate the complex interactions between various mental health symptoms. Network analysis has emerged as a promising new method for exploring the internal relationships among different mental health problems (11, 24–27). Network analysis allows for the identification of influential nodes and the revelation of connections between symptoms of mental health problems in different populations by estimating edge weights between different nodes and calculating node centrality or bridge centrality (28). This approach has been utilized to investigate the interrelationships between mental health problems among medical healthcare practitioners at different stages of the COVID-19 pandemic (8, 29–31). Despite numerous studies on mental health problems among nurses, we know very little about the underlying mechanisms of these problems. The core symptoms of mental health problems faced by psychiatric nurses in the early stages of the pandemic, as well as the interconnections between these symptoms, have rarely been reported. Therefore, it is crucial to employ new explanatory models to gain further insight into the underlying mechanisms of these problems experienced by nurses.

This nationwide survey aims to address this research gap by: (1) identifying core symptoms and bridge symptoms of mental health symptoms among Chinese psychiatric nurses during the early stages of the pandemic using network analysis; (2) exploring the internal relationships between different mental health symptoms in this population; and (3) proposing targeted intervention strategies focused on core symptoms and bridge symptoms to improve overall the mental health of Chinese psychiatric nurses. By employing network analysis, this study seeks to provide novel insights into the complex interplay of mental health issues among psychiatric nurses during a critical period of the pandemic. The findings will contribute to a more comprehensive understanding of the challenges faced by this essential workforce and inform the development of more effective, targeted interventions to support their mental well-being.

## Methods

### Research design and participants

This cross-sectional study was conducted as part of the 2021 National Hospital Performance Evaluation Survey (NHPES), a nationwide survey conducted in China via the WeChat app from January to March 2021. In the early stages of the COVID-19 pandemic, 41 tertiary psychiatric hospitals from 29 provinces were selected as the target hospitals for this national survey. All psychiatric nurses in these target hospitals were invited to participate. Finally, 9,224 psychiatric nurses were included in the statistical analyses, and the response rate was 91.6%.

The socio-demographic variables, including gender, age, marital status, educational level, and the information on the participation of psychiatric nurses in frontline treatment of COVID-19 patients, were collected through an anonymous online questionnaire. The Depression Anxiety Stress Scales-21 (DASS-21) was used to assess depression, anxiety, and stress symptoms. The Maslach Burnout Inventory-Human Service Survey (MBI-HSS) was employed to measure job burnout in the aspects of emotional exhaustion (EE), depersonalization (DP), and personal accomplishment (PA). The research protocol was approved by the Ethics Committee of Chaohu Hospital of Anhui Medical University (approval number: 202002-KYXM-02), and an electronic consent form was obtained from every respondent.

### DASS-21 and MBI-HSS

The DASS-21 is widely recognized and used for assessing depression, anxiety, and stress symptoms in different populations (32–35) and consists of three subscales (depression, anxiety, and stress), each containing 7 items. Each item is scored on a 4-grade Likert scale from 0 (did not apply to me at all) to 3 (applied to me very much or most of the time). The categories for depression scores are as follows: normal (≤ 9 points), mild (10-13 points), moderate (14-20 points), and severe to extremely severe (≥ 21 points). Similarly, the categories for anxiety scores are: normal (≤ 7 points), mild (8-9 points), moderate (10-14 points), and severe to extremely severe (≥ 15 points). Lastly, the categories for stress scores are: normal (≤ 14 points), mild (15-18 points), moderate (19-25 points), and severe to extremely severe (≥ 26 points). The scores for each subscale range from 0 to 42, and increase with levels of depression, anxiety, or stress. The clinically meaningful depression, anxiety, and stress are defined as ≥ 10, ≥ 8, and ≥ 15 points, respectively.

The MBI-HSS scale is primarily used to investigate job burnout in various populations. Because of its good reliability and validity, it has been widely accepted and used by researchers (15, 36–38). The scale consists of 3 subscales: EE, DP, and PA, totaling 22 items, of which the EE subscale consists of 9 items, DP includes 5 items, and PA includes 8 items. Each item on the scale is scored on a scale of 0-6 points. For EE, scores are classified as mild (0-17 points), moderate (18-26 points), and high (≥ 27 points). DP scores are categorized as mild (0-4 points), moderate (5-9 points), and high (≥ 10 points). PA scores are classified as mild (0-33 points), moderate (34-40 points), and high (≥ 41 points). Burnout is defined as EE ≥ 27 points and/or DP ≥ 10 points.

### Statistical analysis

#### Network estimation

The R programming language was employed in the RStudio environment (version 4.3.3) (37), and the Gaussian Graphical Model (GGM) was used to construct network models with the DASS-21 (Depression, Anxiety, and Stress) and MBI-HSS (EE, DP, and PA) (39). In the GGM network model, symptoms were represented as nodes connected by edges (28) with the thickness of the edges in the networks indicating the partial correlation coefficients between the nodes. Thicker edges represent stronger relationships and vice versa (40). Dark blue edges represent positive correlations, while dark red edges indicate negative correlations. The DASS-21 and MBI-HSS network model features 6 nodes, including depression, anxiety, and stress symptoms as assessed by the DASS-21, and EE, DP, and PA symptoms as assessed by the MBI-HSS, generating 15 edges. The network model was estimated by the Spearman correlation, the graphical least absolute shrinkage and selection operator (LASSO) (41), and the Extended Bayesian Information Criterion (EBIC) models (42). Additionally, a penalty parameter of 0.5 was set. The R packages used in the process include mgm, qgraph, bootnet, networktools, and Network Comparison Test (NCT) (43).

#### Network centrality and bridge symptoms

The centrality index is a quantitative measure designed to evaluate the core degree of nodes in a network structure and enables an understanding of the importance of nodes within the entire network. To determine the central nodes in the network, the strength, closeness, betweenness, and expected influence (EI) were calculated. To the extent that the EI of a given node is determined by summing all the edge weights between that node and the other nodes in the network (44), both negative and positive edges surrounding a node were taken into account, providing a measure of the overall positive connectivity in networks. Bridge nodes that connect communities were identified by calculating the bridge expected influence (BEI), which is the sum of the edge weights that connect a given node to all those in the other community and is used to determine the bridge nodes in networks with negative and positive connections (28). The relationships between connected nodes were evaluated through the “qgraph” package (44), while the accuracy and stability of the network edges were determined using the “bootnet” package (40). The “networktools” package was designed to calculate BEI indices, which identify bridging symptoms and illustrate relationships between different symptoms (28).

#### Network stability, accuracy, and network comparison

To evaluate the stability and accuracy of the network, two important measures were devised, i.e. the correlation stability coefficient (CS-coefficient) and the 95% confidence interval (CI) (40). The CS-coefficient evaluates the stability of node strength within the network with Values ≥0.5 indicating high reliability, those between 0.25 and 0.5 moderate reliability, and those <0.25 low network robustness. To determine the edge accuracy, nonparametric bootstrapping was used to estimate the 95% CIs of the edge weights. A wider CI indicates lower accuracy and reliability in estimating edges, while a narrower CI implies a more reliable network that estimates edge weights with greater accuracy. Additionally, to compare network models based on gender and participation in frontline work treating COVID-19 patients, the NCT was used to directly compare the network models of different dimensions, including the Independent Groups Gaussian Network Comparison Test and the Global Strength Invariance Test (45).

## Results

### Socio-demographic characteristics of psychiatric nurses in China

The socio-demographic characteristics of Chinese psychiatric nurses were shown in **Table 1**. A total of 9,224 Chinese psychiatric nurses were included in this study, aged 19 to 60 years (35.3 ± 8.6) of whom 7,305 (79.2%) were female, 6,774 (73.4%) were married, and 6,422 (69.6%) held a college degree. Additionally, 20.4% (1,885/9,224) of the participants had ever participated in frontline treatment of COVID-19 patients. Overall, 27.6% of the participants fell into the category of having clinically meaningful depression, while 31.2% had anxiety and 14.5% had stress. Of all the participants in this research, 17.4% (1,604) reported experiencing moderate to extremely severe depression. In addition, 23.3% (2,268) reported experiencing moderate to extremely severe anxiety, while 7.4% (681) experienced moderate to extremely severe stress. The percentages of moderate to extremely severe depression, anxiety, and stress among females were 8.7% (636), 17.8% (1,302), and 7.2% (525), respectively. Among those suffering from clinically meaningful anxiety, 25.9% (456) had participated in frontline treatment of COVID-19 in the previous year. According to the MBI-HSS scale, the prevalence of EE, DP, and job burnout was 13.4%, 20.5%, and 23.8%, respectively. DP was higher in males (7.04 ± 6.28) compared to females (6.15 ± 5.55) (z = -4.515, p < 0.001), and females accounted for 73.7% of the total number of people suffering from DP.

**Table 1:** Demographic characteristics of 9,224 Chinese psychiatric nurses.

Variables	N (%)	Mean (SD) or Median (IQR)
Age (years)		35.3 (8.6)
Sex		
Male	1919 (20.8)	
Female	7305 (79.2)	
Marital status		
Single	2064 (22.4)	
Married	6774 (73.4)	
Divorced or widowed	386 (4.2)	
Education level		
Associate degree or less	2712 (29.4)	
College degree	6422 (69.6)	
Master’s degree or more	90 (1)	
In the frontline work of COVID-19	1885 (20.4)	
DASS-21		
DEP ^a^	2549 (27.6)	4.0 (10.0)
Normal	6675 (72.4)	0 (4.0)
Mild	945 (10.2)	10.9 (1.0)
Moderate	1233 (13.4)	15.3 (1.9)
Severe and extremely severe	371 (4.0)	28.2 (5.9)
ANX ^b^	2875 (31.2)	4.0 (8.0)
Normal	6349 (68.8)	2.0 (4.0)
Mild	607 (6.6)	8.0 (0)
Moderate	1545 (16.7)	12.1 (1.7)
Severe and extremely severe	723 (7.8)	20.9 (5.8)
STR ^c^	1336 (14.5)	7.9 (7.5)
Normal	7888 (85.5)	5.7 (5.0)
Mild	655 (7.1)	16.7 (1.0)
Moderate	393 (4.3)	21.5 (1.6)
Severe and extremely severe	288 (3.1)	30.8 (4.8)
MBI-HSS ^d^	2196 (23.8)	
EE ^e^	1240 (13.4)	14.0 (11.5)
DP ^f^	1894 (20.5)	6.3 (5.7)
PA ^g^	3519 (38.2)	28.3 (12.6)

DEP, Depression; ANX, Anxiety; STR, Stress; EE, Emotional Exhaustion; DP, Depersonalization; PA, Personal Accomplishment; ^a^ clinically meaningful depression ≥ 10; ^b^ clinically meaningful anxiety ≥ 8; ^a^ clinically meaningful stress ≥ 15; ^d^ clinically meaningful burnout (EE ≥ 27 or DP ≥10); ^e^ EE ≥ 27; ^f^ DP ≥10; ^g^ PA>33.

### Estimation of the network and centrality of the relationship between burnout and psychological symptoms

**Figure 1:**
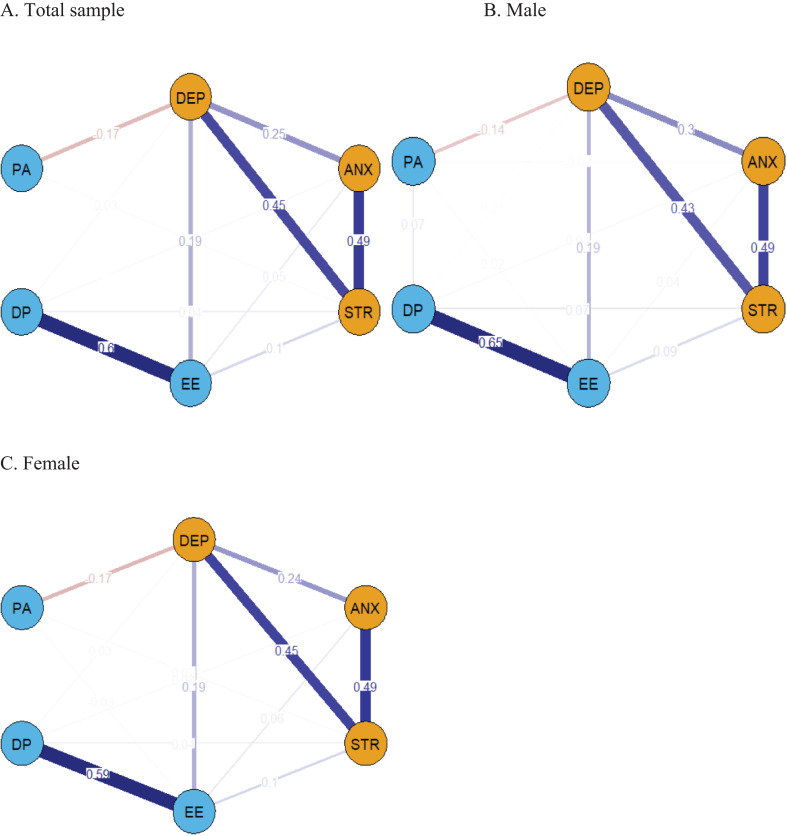
The network structure between the three subscales in the DASS-21 and MBI-HSS questionnaire. Positive connections are graphically depicted with dark blue lines, while negative connections are shown with dark red lines. The thickness of the edges indicates the strength of the correlation. Nodes with stronger correlation tend to be closer together spatially. DEP, Depression; ANX, Anxiety; STR, Stress; EE, Emotional Exhaustion; DP, Depersonalization; PA, Personal Accomplishment. **(A)** represents the network structure of the population sample, while **(B)** represents the network structure of the male sample, and **(C)** represents the network structure of the female sample.

**Figure 1A** illustrates the network structure encompassing depression, anxiety, stress, and physician burnout. Within the comprehensive network, 13 out of the possible 15 connections (86.7%) showed nonzero values, indicating substantial interconnectivity among the symptoms with the mean weight of 0.138 indicating the overall strength of the connections within the network. The individual nodes featured a predictability range from 0.060 to 0.772, averaging 0.602, as shown in **Table 2**, indicating that, on average, 0.602 of the variability in each node could be attributed to its adjacent nodes. The stress, depression, and anxiety subscales among DASS-21 demonstrated good predictability, with values of 0.772, 0.758, and 0.742, respectively. However, the MBI-HSS questionnaire demonstrated the lowest predictability for personal accomplishment, with a value of 0.060. The most significant associations were found between EE and DP, anxiety and stress, and depression and stress, with correlation coefficients of 0.602, 0.488, and 0.446, respectively.

**Table 2:** Centrality plots for EBICglasso network depicting the betweenness, closeness and EI of each node (variable).

Abbr.	Betweenness ^f^	Closeness ^f^	Strength ^f^	EI ^f^	Predictability ^f^	Predictability (Male)	Predictability (Female)
EE	0.598	-0.119	0.405	0.558	0.676	0.729	0.662
DP	-0.598	-0.740	-0.303	0.024	0.605	0.673	0.586
PA	-0.598	-1.190	-1.829	-1.932	0.060	0.061	0.061
DEP	1.793	1.593	0.816	0.129	0.758	0.779	0.753
ANX	-0.598	-0.208	0.057	0.303	0.742	0.766	0.735
STR	-0.598	0.664	0.855	0.920	0.772	0.811	0.763

f the values are raw data from the total sample. EE, Emotional Exhaustion; DP, Depersonalization; PA, Personal Accomplishment; DEP, Depression; ANX, Anxiety; STR, Stress.

**Figure 2:**
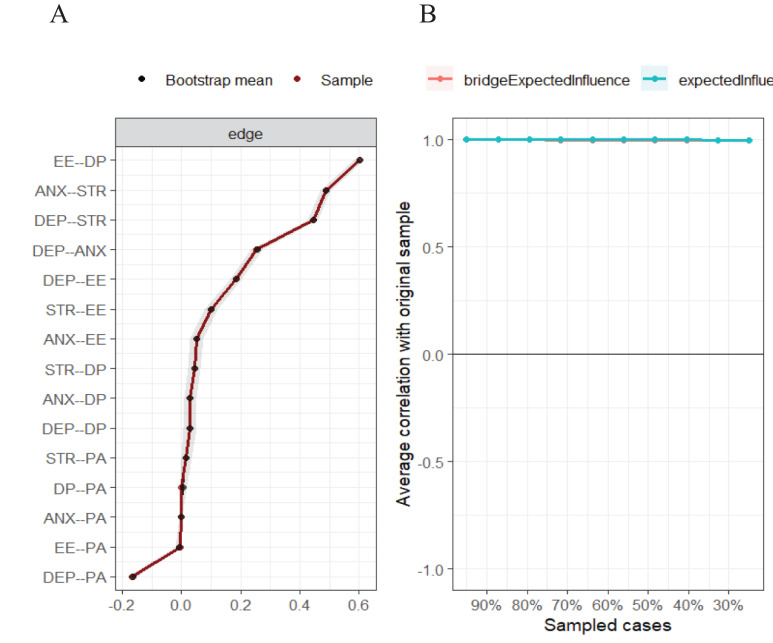
Accuracy and stability of the network. **(A)** Accuracy of edges estimation in total sample; **(B)** Centrality stability was tested using the case-dropping bootstrap method. The horizontal axis represents the proportion of samples participating in the test in relation to the total sample size, while the vertical axis represents the average similarity to the original sample.

**Figure 3:**
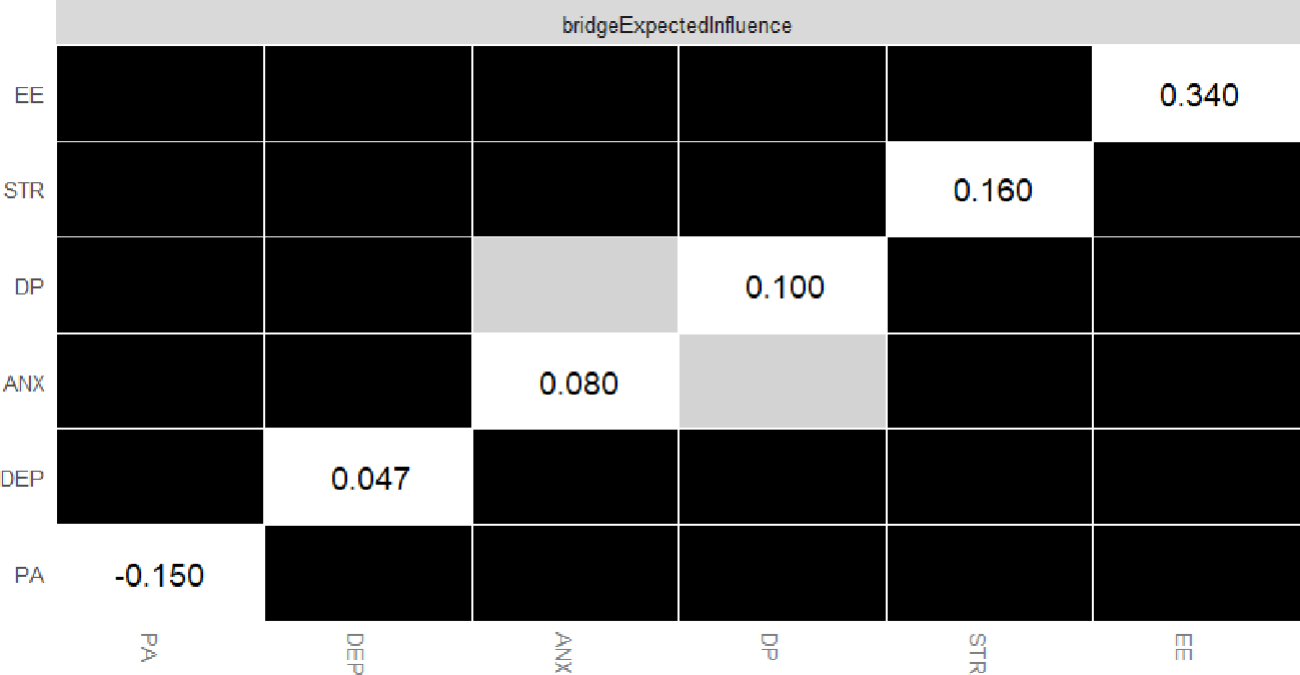
The bootstrap edge weights difference test of the node’s strength in the network. The black boxes indicate significant differences in strength between two variables. The grey boxes represent non-significant differences.

**Table 2** reveals the related parameters of the network, including betweenness, closeness, strength, and EI. Stress had the highest EI value of 0.920, indicating its dominant role in the network model, followed in order by EE, anxiety, depression, and DP. PA had the least impact on the network structure. Node stress had the highest strength among all nodes, while node depression had the highest scores for both closeness and intermediation index (betweenness). The intergroup association between EE and depression was found to be the strongest (**Figure 2A**). In terms of bridging symptoms, EE had the highest BEI of 0.340 (**Figure 3**), indicating that EE plays a key role in linking various clusters of symptoms.

### Accuracy and stability of the network

The network demonstrates high accuracy and stability, which can be evidenced by comparing the average associations obtained through resampling (bootstrap averages) (**Figure 2**). **Figure 2A** shows the accuracy of edge estimation in the total sample. The convergence of the black and red lines indicates satisfactory accuracy, while the narrow gray band suggests minimal variability during resampling. **Figure 2B** displays the results obtained from the centrality stability test through the case-dropping bootstrap method, which focuses on the stability of edge weights. The results of nonparametric bootstrapping demonstrate accurate edge estimation along with narrow confidence intervals. The case-dropping bootstrapping also yielded CS-coefficients of 0.75 for both BEI and EI, indicating stable results above 0.5.

### Network comparison by gender and by whether to participate in frontline treatment of COVID-19 patients

**Figures 1B, C** represent the network structures of the male and female samples, and no statistically significant difference between the male and female networks was shown through the Gaussian network comparison test of independent groups (Female: 7305 vs Male: 1919; M: 0.072, p=0.079). The global strength invariance test for male and female networks, however, showed a statistically significant difference (S: 0.113, p=0.020), indicating that the sum of the absolute values of the weights of all edges in the two networks is significantly different. In accordance with the network structure data for males and females, stress has the highest strength and EI, while depression features the highest closeness, betweenness, and the strongest bridging strength. EE shows the highest BEI, and the EE-DP edge exhibits the highest intensity. The strength and EI of DP in males, however, are significantly higher than those in females (all, p < 0.05). There was no significant difference between the independent groups Gaussian network comparison test (M: 0.042, p=0.644) and the global strength invariance test (S: 0.033, p=0.574) regarding the participation in frontline treatment of COVID-19 patients.

## Discussions

Our results showed a high prevalence of anxiety, depression, stress, and burnout among psychiatric nurses, and we also examined their interconnections. Notably, stress, with its highest expected influence value, stands out as the most impactful symptom, suggesting that it may play a pivotal role in the onset or exacerbation of other mental health issues. Depression’s significant position as a bridging node indicates its crucial role in the interconnectedness of symptoms, highlighting the potential benefits of targeted interventions. The absence of significant differences across gender and frontline work experience points towards a universal impact of these stressors, further emphasizing the need for comprehensive strategies to address these mental health concerns across the board. As primary caregivers for patients, including COVID-19 patients, nurses are at a greater risk of infection and face mental health problems (18, 19, 21, 22). Although research has extensively examined the connections between depression, anxiety, and stress, few studies have examined the interconnections between burnout and other mental health symptoms (17, 46). Our study addresses this gap by employing network analysis to explore the intricate relationships between depression, anxiety, stress, and burnout among Chinese psychiatric nurses during the early stages of the pandemic.

Our study showed that 27.6% of participants experienced clinically significant depression, 31.2% reported anxiety, and 14.5% encountered stress. Additionally, the prevalence rates of EE, DP, and overall job burnout were 13.4%, 20.5%, and 23.8%, respectively. These figures are comparable to the findings from a meta-analysis of 401 studies, which reported depression rates of 28.5%, anxiety at 28.7%, post-traumatic stress disorder at 25.5%, and insomnia at 24.4%. Furthermore, this meta-analysis pointed out the fact that women have significantly higher odds of mental health problems (47). A survey conducted among 54,025 nurses in the U.S. reports that over half of the respondents experienced emotional drain (50.8%), feeling used up (56.4%), and burnout (45.1%) several times a week or daily during the pandemic (48). Our data also indicate that psychiatric nurses in China experienced a lower incidence of job burnout (overall 23.8%, EE 13.4%, DP 20.5%) compared to emergency nurses, whose burnout rates ranged from 48.1% to 53.3% (49, 50). The discrepancy in burnout prevalence between emergency and psychiatric nurses is likely attributable to their distinct professional roles. Emergency nurses experience higher work intensity and greater stress (49), whereas psychiatric nurses tend to have high levels of depression, anxiety, stress, and burnout in this study. This suggests the need for protective or preventative measures and targeted interventions to mitigate the mental health impact on psychiatric nurses, addressing both core symptoms and broader burnout issues.

In our survey, we found a significantly higher rate of anxiety (31.2%) in psychiatric patients compared to the general public (15.2-25.0%) (51, 52). Previous studies have shown a connection between anxiety and other mental health issues, including suicidal ideation and insomnia (26, 53, 54). Oliva et al.’s research (2024) suggests that anxiety has a significant impact on suicidal ideations, second only to depression. Interestingly, this study found that anxiety has the highest prevalence, while the stress of psychiatric nurses has the highest EI value and the highest strength among all nodes, indicating that stress is the most central symptom of mental health problems among psychiatric nurses. **Figure 2A** also illustrates that anxiety is strongly associated with stress, as consistent with other studies (26), and these findings could potentially suggest that stress is the underlying cause of anxiety in psychiatric nurses. More evidences suggest that distinct characteristics of mental health problems vary with the stages of the pandemic regardless of population (55, 56). In the early stage of the COVID-19 pandemic, anxiety symptoms may emerge as the primary manifestation of mental health problems among different populations (56). However, suicidality, posttraumatic stress symptoms, and depression consistently increased among different populations during the later stage of the pandemic (8, 57–59). Based on our survey results, it is speculated that psychiatric nurses are under higher levels of anxiety during the early stages of the pandemic, which may be attributed to the stress caused by their heavy workload and concern about the unknown viruses. Therefore, it may help alleviate their anxiety during the pandemic to provide effective interventions to psychiatric nurses who are under high levels of stress.

This study indicates that the node “depression” has the highest scores for both closeness and betweenness, and EE had the highest BEI. The intergroup association between EE and depression was found to be the strongest, consistent with previous studies (49). Huang et al.’s study (2024) revealed that 54.6% of participants displayed depressive symptoms, while 48.1% experienced severe job burnout. Moreover, approximately 37.1% of the variance in depression was caused by the components of job burnout (49), which have continuously increased among healthcare workers throughout the pandemic (23, 60) with PTSD as a possible culprit (15, 61). The strong connection between depression and EE offers a theoretical basis for determining which intervention measures to be implemented, and the connection between mental health problems and burnout is complex and varied, highlighting the importance of further research to explore the socio-psychological mechanisms. To the extent that mental health problems and burnout in healthcare workers have attracted attention from the authorities (62), it is highly recommendable to launch programs to enhance the mental health of healthcare workers, such as trauma-related interventions, cognitive-behavioral therapy, and physical activity (63–67). Due to the accessibility and ease of practice, it should be prioritized as a complementary intervention in preventing and treating mental health problems among healthcare practitioners (68–70).

The global strength invariance test for male and female networks showed a statistically significant difference, indicating that the sum of the absolute values of the weights of all edges in the two networks differs significantly. It is shown in the centrality invariance test that the strength and EI of DP are the reasons for the difference between the “gender” networks, and DP is more severe in males and more prevalent among females. Many studies have demonstrated that there are gender differences in mental health problems during the pandemic (71–73). Consistent with our findings, Rusandi et al.’s study (2022) showed that the burnout variable for the Exhaustion and Professional Efficacy indicators was higher in females than in males, and male healthcare workers with close contact with COVID-19 patients experienced an increase in depersonalization (60). However, no significant statistical differences in the independent groups Gaussian network comparison test are found among the networks of “whether to participate in frontline treatment of COVID-19 patients” and “gender”. This is probably because the short-term mental health consequences of COVID-19 were equally severe among affected countries and genders (52). Most nurses are female and thus more vulnerable to the adverse effects of the pandemic for they have to navigate challenges in their personal lives and families in addition to enormous stress at work. Therefore, it is crucial to call attention to and intervene in women’s mental health issues to effectively address the gender inequity exacerbated by the pandemic (13, 74).

Several limitations of this study should be acknowledged. Firstly, the cross-sectional design confines our ability to investigate causal relationships between different symptoms. Secondly, the data used in the analysis were collected during the early stages of the COVID-19 epidemic, with no data available from the later stages. Therefore, we were unable to analyze the dynamic changes in burnout and emotional symptoms throughout the entire course of the epidemic. Finally, due to the anonymous nature of the survey, we were unable to provide individual intervention and treatment to these participants.

## Conclusions

In summary, our results revealed that burnout, depression, anxiety, and stress are relatively common among psychiatric nurses during the COVID-19 pandemic. While anxiety was the most prevalent issue, stress was identified as the core symptom. Depression was identified as a crucial bridging node, highlighting its potential role in the development and maintenance of other mental health issues. EE, a key component of burnout, showed the strongest association with depression, emphasizing the close relationship between these two constructs. Our findings underscore the need for targeted interventions that address the core symptoms and bridging nodes identified in this study. By focusing on stress reduction techniques and strategies to manage depression, healthcare organizations may more effectively support the mental well-being of psychiatric nurses during and beyond the pandemic.

## Data Availability

The original contributions presented in the study are included in the article/supplementary material. Further inquiries can be directed to the corresponding author/s.
